# Effects of an emotional intelligence program in variables related to the prevention of violence

**DOI:** 10.3389/fpsyg.2015.00743

**Published:** 2015-06-01

**Authors:** Maite Garaigordobil, Ainize Peña-Sarrionandia

**Affiliations:** Department of Personality, Assessment, and Psychological Treatments, Faculty of Psychology, University of the Basque CountryDonostia-San Sebastián, Spain

**Keywords:** emotional intelligence, violence, intervention, adolescence, gender

## Abstract

In recent decades, numerous studies have shown a significant increase in violence during childhood and adolescence. These data suggest the importance of implementing programs to prevent and reduce violent behavior. The study aimed to design a program of emotional intelligence (EI) for adolescents and to assess its effects on variables related to violence prevention. The possible differential effect of the program on both genders was also examined. The sample comprised 148 adolescents aged from 13 to 16 years. The study used an experimental design with repeated pretest–posttest measures and control groups. To measure the variables, four assessment instruments were administered before and after the program, as well as in the follow-up phase (1 year after the conclusion of the intervention). The program consisted of 20 one-hour sessions. The pretest–posttest ANCOVAs showed that the program significantly increased: (1) EI (attention, clarity, emotional repair); (2) assertive cognitive social interaction strategies; (3) internal control of anger; and (4) the cognitive ability to analyze negative feelings. In the follow-up phase, the positive effects of the intervention were generally maintained and, moreover, the use of aggressive strategies as an interpersonal conflict-resolution technique was significantly reduced. Regarding the effect of the program on both genders, the change was very similar, but the boys increased assertive social interaction strategies, attention, and emotional clarity significantly more than the girls. The importance of implementing programs to promote socio-emotional development and prevent violence is discussed.

## Introduction

More than 1.3 million people die each year as a result of violence (World Health Organization [WHO], 2014). In the Basque country, in the past year, 83.7% of adolescents have been involved in situations of bullying, and 69.8% in cyberbullying (Garaigordobil, 2013). Currently, problems like bullying, racism, sexism, loneliness, depression, etc., are common and all of them are related to social and emotional skills. Is there any solution? Which one? A possible response is to develop emotional intelligence (EI).

Studies examining the relationship between aggressive behavior and EI are scarce (Inglés et al., 2014). Still, these studies show that emotional attention is positively related to anger, and that greater clarity and emotion repair are related to lower trait/state anger, lower internal expression of anger, and higher anger control (Salguero and Iruarrizaga, 2006). In addition, people with a high emotional clarity better understand the emotions they are feeling, their causes, and consequences. Finally, people with high EI resolve conflicts more constructively (Zeidner and Kloda, 2013) and display fewer aggressive behaviors (García-Sancho et al., 2014). Thus, adolescents with more emotional skills present fewer negative emotions related to the expression of aggressive behavior, such as anger and hostility (Extremera and Fernández-Berrocal, 2013).

Recent studies have shown that programs to improve EI (Qualter et al., 2007; Di Fabio and Kenny, 2011) also increase positive leadership (Muñoz de Morales and Bisquerra, 2013) and decrease aggressiveness and anger (Castillo et al., 2013). Regarding socio-emotional intervention programs, it has been confirmed that such programs improve self-control of behavior (Choque-Larrauri and Chirinos-Cáceres, 2009), assertive behaviors (Melero and Palomera, 2011), personal and social problem-solving (Maurer et al., 2004), the ability to analyze one’s feelings (Garaigordobil, 2008), and reduce aggressiveness (Frey et al., 2005).

Regarding gender, although various studies found no differential effects of changes in EI in boys and girls (Eisen et al., 2003; Kimber et al., 2008; Ogunyemi, 2008; Choque-Larrauri and Chirinos-Cáceres, 2009), others found that boys improve EI (Melero and Palomera, 2011) and empathy level (Castillo et al., 2013) more than girls, and still another study reported that girls in general improved their scores significantly more than boys (Byrne et al., 2004).

These discrepancies could be explained by the possible differences between boys and girls before the intervention on the dependent variables assessed in the diverse investigations. For example, boys tend to obtain lower scores in empathy (e.g., Garaigordobil, 2013), and this may be related to differences in their socialization process, as girls’ education is more focused on emotions, whereas boys are taught to minimize certain emotions (Sánchez-Núñez et al., 2008) and they conclude that they must be strong, practical and unemotional.

The theoretical framework of this study is based on the model of Mayer and Salovey (1997), in which EI is made up of four dimensions: perception, facilitation, comprehension, and emotion regulation. Perceiving Emotions refers to the ability to be aware of emotions in oneself and others as well as in objects, art, stories, music, and other stimuli. Facilitating thought is the ability to generate, use, and feel emotion as necessary to communicate feelings or employ them in other cognitive processes. Understanding emotions consists of the ability to understand emotional information, how emotions combine and progress through relationship transitions, and to appreciate such emotional meanings. Emotion regulation is the ability to be open to feelings, and to modulate them in oneself and others so as to promote personal understanding and growth. EI can be understood as a set of cognitive-emotional skills, as well as a personality trait. The study aimed to design a program of EI for adolescents and to assess its effects on variables related to violence prevention.

If the program teaches adolescents to moderate their negative emotions, intensifying the positive ones, internally control their feelings of anger, increase their knowledge of assertive strategies for coping with problematic social situations, or become more aware of the harmful consequences of negative emotions for themselves and for others, then their violent behavior is expected to decrease. In addition, previous studies have shown that these variables that are expected to be positively influenced by the program are associated with violent behavior (Garaigordobil, 2013) and that there are significant positive correlations between assertive cognitive strategies and empathy, as well as between aggressive cognitive strategies and anti-social behavior (Garaigordobil, 2008).

The results of other studies point in the same direction. Swearer et al. (2014) consider that to reduce bullying, interventions must address psychological, cognitive, and social factors. In fact, bullying prevention and intervention programs should teach students skills to promote effective problem-solving strategies. Cognitions supporting bullying and beliefs about positive versus negative consequences affect the likelihood of adolescents becoming bullies. Thus, interventions focusing on cognitive and social functioning are important to break the cycle of bullying involvement. Jones et al. (2012) also confirm that effective bullying prevention programs should consider implementing social-emotional learning programs, which teach youth skills such as emotion management, communication skills, empathy, problem solving, etc.

To design the intervention program assessed in the present investigation, we took into account previous works (Di Fabio and Kenny, 2011; Castillo et al., 2013) with regard to the theoretical framework, the methodology of the study, and the characteristics of the activities contained in the intervention proposal.

The objectives of Di Fabio and Kenny’s (2011) program, carried out with adolescents aged 16–18 years, were: (1) To improve the ability to perceive emotions in oneself and others; (2) To develop the ability to generate, use, and feel emotion to facilitate cognitive processes; (3) To develop the ability to understand emotional information, and (4) To improve the ability to cope with emotions. The training, based on the model of Mayer and Salovey (1997), was subdivided into four weekly sessions of 2½ h each. The study used a quasi-experimental design with repeated pretest–posttest measures and control groups. The program improved the participants’ EI (appraisal and expression of emotions, emotion perception and regulation, emotion utilization, etc.).

The program to promote EI of Castillo et al. (2013) had four goals: (1) accurate perception, appraisal, and expression of emotions; (2) awareness of feelings and ability to generate emotions to facilitate thought; (3) understanding of emotions, including the ability to label them with a rich emotional vocabulary; and (4) regulation of emotions in order to promote emotional and intellectual growth. The EI training lasted 2 years and involved 12 sessions for each academic year. The study used a quasi-experimental pretest–posttest design with a control group. The results showed that the students in the experimental group displayed lower levels of aggressiveness, anger, hostility, and personal distress, and higher levels of empathy.

Drawing on the above, the present study had the main goal of assessing the effect of the program on EI, cognitive strategies to resolve social situations, feelings of anger, and the ability to cognitively analyze negative emotions. The possible differential effect of the program on both genders was also examined.

Taking into account the results obtained in previous works showing the effectiveness of some interventions to improve EI (Qualter et al., 2007; Di Fabio and Kenny, 2011; Castillo et al., 2013), as well as the positive impact of programs of social-emotional development on the ability to analyze negative feelings (Garaigordobil, 2008), or on assertiveness (Melero and Palomera, 2011), six hypotheses are postulated: (H1) the program will improve EI (attention, clarity, emotional repair); (H2) the program will increase assertive cognitive social interaction strategies, decreasing aggressive and passive strategies; (H3) the program will decrease feelings of anger (state-anger, trait-anger) and its expression in anger situations, increasing control over such feelings (anger control-out, and anger control-in); (H4) the program will improve the cognitive capacity to analyze negative feelings (anger, envy, sorrow, fear), that is to say, to identify causes (factors, situations, or stimuli that generate these emotions), consequences (the impact of those emotions at the behavioral, cognitive, and emotional level), and ways to resolve these emotions (behavioral, cognitive, and emotional coping strategies to constructively resolve negative emotions); (H5) the program will affect both genders similarly; and (H6) the effects of the program will be stable 1 year after the intervention.

## Materials and Methods

### Participants

This study was conducted with a sample of 148 Spanish adolescents aged between 13 and 16 years, of whom 83 (four groups) were randomly assigned to the experimental condition and 65 (three groups) to the control condition. The initial sample included 161 participants, but due to experimental mortality, 13 were excluded (for incorrect completion of instruments or the adolescents’ absence at posttest). Of these 148 participants, 45.3% were boys (*n*= 67) and 54.7% were girls (*n*= 81). The adolescents studied third course of Compulsory Secondary Education (Grade 9). No statistically significant differences were found in the socio-demographic characteristics of the experimental and control adolescents, taking into account gender, age, type of school, and parents’ level of education (see **Table 1**). One year after the intervention had been carried out, a follow-up phase was performed, observing a sample mortality of 18 participants (due to changes in these students’ schools).

**Table 1:** Socio-demographic characteristics of the experimental and control sample.

		Experimental *F*(%)	Control *F*(%)	χ^2^	*p*
Gender	Boys	37 (44.6)	30 (46.2)	0.037	0.848
	Girls	46 (55.4)	35 (53.8)		
Age	13	19 (22.9)	11 (16.9)	2.935	0.402
	14	58 (62)	44 (67.7)		
	15	5 (6)	8 (12.3)		
	16	1 (1.2)	2 (3.1)		
Type of school	Public	60 (72.3)	43 (66.2)	0.65	0.421
	Private/subsidized	23 (27.7)	22 (33.8)		
Father’s educational level	Primary level studies	3 (4.0)	3 (4.9)	4.68	0.321
	Secondary level studies	28 (37.3)	13 (21.3)		
	Higher secondary studies	26 (34.7)	23 (37.7)		
	Qualified/diploma	10 (13.3)	12 (19.7)		
	Graduated	8 (10.7)	10 (16.4)		
Mother’s educational level	Primary level studies	3 (4.1)	5 (7.8)	1.39	0.846
	Secondary level studies	21 (28.4)	16 (25)		
	Higher secondary studies	17 (23)	13 (20.3)		
	Qualified/diploma	26 (35.1)	22 (34.4)		
	Graduated	7 (9.5)	8 (12.5)		

*F*, frequency; (%), percentage; χ^*2*^, Pearson chi square; *p*, significance.

The selection of schools was random, incorporating public and private centers that represent different socio-economic-cultural levels. The list of schools of the Education Department of the Basque Government was used to select the sample. The centers of the list were categorized according to the socio-economic-cultural level (low–medium–high). Using a simple random design, one center from each category was selected and a letter inviting its collaboration was sent. When a center declined to participate, another center of the same category was randomly selected and the procedure was repeated.

### Design and Procedure

The study used a quasi-experimental design with repeated pretest–posttest measures and control groups. Regarding the procedure, the following phases were conducted: (1) a letter was sent to the headmasters of randomly selected schools from the list of centers in Bizkaia with an explanation of the project and a request for their collaboration; (2) with those who agreed to collaborate, we conducted an interview to present the project and deliver the informed consent forms for the parents; (3) four pretest assessment instruments were subsequently administered; (4) the intervention program was carried out in the experimental groups while the control groups received a regular tutorship program of their center; (5) after the intervention, at posttest, the same instruments as at pretest were administered to the experimental and control groups; and (6) one year after the intervention, a follow-up phase took place, administering the same instruments once more. The study respected the ethical values required in research with human beings (informed consent, confidentiality, etc.), having received a favorable report from the Ethics Committee of the University of the Basque Country (CEISH/146/2012).

### Assessment Instruments

In order to assess the effects of the intervention before and after the program, we administered four assessment instruments with psychometric guarantees of reliability and validity.

*Trait Meta-Mood Scale* (*TMMS-24*; Salovey et al., 1995; Spanish adaptation of Fernández-Berrocal et al., 2004) assesses intrapersonal EI using three factors: attention, clarity, and emotional repair. Attention to Feelings is the amount of attention paid to one’s emotional states. This subscale assesses a basic ability in meta-mood experience referred to the tendency to take notice of and value mood. Emotional Clarity refers to understanding one’s emotional states. This subscale concerns the extent to which people experience their feelings clearly or understand how they feel. It is a relatively enduring tendency to monitor one’s feelings and to experience them lucidly. Emotional Repair is the ability to regulate one’s emotional states. It refers to the individual’s belief about his/her ability to quit and regulate negative emotional states and to extend positive ones. The scale consists of 24 statements about which respondents rate their degree of agreement on a 5-point Likert scale ranging from 1 (*strongly disagree*) to 5 (*strongly agree*). The scale has shown high internal consistency both in the version of Fernández-Berrocal et al. (2004; α > 0.85 in all the scales) and in our study with α = 0.94 for the total score and between 0.86 and 0.89 for the subscales. The Spanish adaptation of the TMMS-24 showed good convergent and discriminant validity, finding positive correlations between Clarity and Emotional Repair and life satisfaction and negative correlations with depression and rumination, as well as positive correlations between Attention and depression and rumination.

The “Cuestionario de Estrategias Cognitivas de Resolución de Situaciones Sociales” (EIS; in English, Cognitive Strategies for Resolution of Social Situations Questionnaire; Garaigordobil, 2008) was used to explore the cognitive strategies to solve six conflictive social situations in which respondents should: address a moral conflict, respond to an aggression, make friends, cope with others’ rejection, recover an object that has been taken by another peer, and resist pressure (e.g., refuse drugs). The adolescents report all the resolution strategies that the protagonist can perform in each situation. The test assesses assertive, passive, and aggressive social interaction strategies. Assertive strategies are those in which the problem situation is directly faced, and effective behaviors are proposed to achieve the goal non-aggressively (developing ability, asking, expressing one’s feelings, asserting one’s rights, dialog and reasoning with the other, etc.). Passive strategies do not directly address the problem situation, and responses of inhibition (there is no action), submission, avoiding the situation, or seeking help from others are proposed for its resolution. Aggressive strategies include responding with behavior that is negative or aggressive for the interaction (threats, physical and verbal aggression, insults, blackmail, reporting others so they will be punished, etc.). The reliability of the questionnaire is acceptable both in the original (α = 0.72, 0.74, and 0.68, for assertive, passive, and aggressive strategies, respectively) and in the present study (α = 0.72, 0.72, and 0.68). The results support the convergent and divergent validity of the instrument, showing that adolescents who used many assertive cognitive strategies as techniques to resolve social conflicts had a positive self-concept and capacity for empathy, displaying few withdrawal behaviors and few antisocial behaviors.

State-Trait Anger Expression Inventory in Children and Adolescents (STAXI-NA; Del Barrio et al., 2005) assesses state/trait anger, its expression (internal and external), and control (external and internal). State-Anger: level of feelings of anger at the time of assessment; Trait-Anger: feelings of anger and frustration habitually experienced, tendency to react frequently with rage and fury. Anger Expression-Out (expression of anger toward other persons or objects); Anger Expression-In (holding in or suppressing angry feelings); Anger Control-Out (controlling angry feelings by preventing their expression toward persons or objects); and Anger Control-In (controlling feelings of anger by calming down or cooling off). The reliability of the instrument in all four dimensions (α between 0.53 and 0.89) was generally adequate, although it was low for external expression of anger. The internal consistency obtained in the present study sample was adequate for the total test (α = 0.74). The analyses confirm the convergent and discriminant validity of the STAXI-NA, finding that feelings of anger have high correlations with aggressiveness, while consideration for others and self-control correlate positively with anger control, and withdrawal correlates positively and significantly with state-anger, trait-anger, and anger expression.

“Cuestionario de evaluación de la capacidad de análisis de sentimientos” (CECAS; in English, Questionnaire for the assessment of the ability to analyze feelings; Garaigordobil, 2008) is a questionnaire used to explore the cognitive ability of analysis of four negative emotions: sadness, envy, anger, and fear. The task requests respondents to identify causes (factors, situations, or stimuli that generate these emotions), consequences (the impact of those emotions at the behavioral, cognitive, and emotional level), and ways to resolve these emotions (behavioral, cognitive, and emotional coping strategies, to constructively resolve negative emotions). For example, with regard to sad feelings, they suggest as causes “the death of a close friend or relative or failing exams,” as consequences “isolation or aggressiveness,” and as ways of dealing with them “talking to friends or studying more for the next exam.” The reliability of the test is good both in the original study (α = 0.75) and in this study (α = 0.92). In addition, validation studies found that adolescents who were able to provide a large number of answers about the causes of feelings showed few withdrawal behaviors, many prosocial behaviors, had a good self-concept, high capacity of empathy, etc. Likewise, adolescents who listed many positive ways of resolving negative emotions also displayed many hetero-assertive behaviors, many cognitive strategies to resolve problematic social situations, and they were nominated by their peers as prosocial individuals.

### The Intervention Program

The program aims to develop EI during adolescence. The intervention consisted of 20 h-long sessions, carried out weekly during a school year (2012–2013). The program was implemented by a Psychology Master researcher, although it can be applied by a teacher, a psychologist, or a psycho-pedagogue. The program is made up of 31 activities distributed in five modules (see **Table 2**): self-awareness, emotion regulation, mood, communication, and empathy.

**Table 2:** Program modules and activities.

Modules	Activities
(1) Self-awareness	(1) Mental gymnastics
	(2) Collage and dramatization
	(3) Laugh at yourself
	(4) Range of feelings
	(5) That’s what I’m like
	(6) Who am I?
(2) Emotion regulation	(7) Emotional perception
	(8) Observers
	(9) Regulating emotions
	(10) Changing beliefs
	(11) Music to regulate emotions
(3) General mood	(12) The thousand and one nights
	(13) Exchanging affection
	(14) Dancers
	(15) The bag
	(16) Feeling better
	(17) Happiness
(4) Communication	(18) Emotions trivial pursuit
	(19) Active listening and assertiveness
	(20) Non-verbal communication
	(21) Assertiveness
	(22) Body language
	(23) Communication skills
	(24) Communication and assertiveness
(5) Empathy	(25) Walking
	(26) Step into another’s shoes
	(27) Secrets
	(28) A picture, a thousand emotions
	(29) Stories
	(30) Questions and answers
	(31) Empathy

An example of activity is “Body Language,” which aims to: (1) identify, understand, and express emotions; and (2) learn to solve social conflicts. For this purpose, two activities are carried out. The first is to brainstorm about emotions. From all emotions proposed by the students, the teacher assigns an emotion to each student, and the adolescents should walk around the classroom expressing this emotion non-verbally until they find the classmate who has been assigned the same emotion. In the second activity, students play in small groups where they will see different images and should indicate the expressed emotion, its causes, consequences, and ways of resolving these emotions. The debate raises questions about the interactions and emotions experienced (e.g., is it to easy identify emotions; what have you learned; how did you feel; etc.).

The application of the program to a group implies four constant variables that make up the methodological framework of the intervention (Garaigordobil, 2008): (1) inter-session constancy (i.e., performing a weekly 1-h session); (2) spatial–temporal constancy (i.e., the program is applied on the same week day, at the same time, and in the same physical space); (3) constancy of the adult program director (i.e., a psychopedagogue or one with such training); and (4) constancy in the session structure.

The training sessions are structured in the following way: (1) the adolescents sit in a large circle, and the adult explains the activity, its goals, and the instructions; (2) the group members carry out the action; and (3) the activity ends with the adolescents sitting on the floor in a circle, discussing the session through guided reflection with the adult who directed the intervention. In the discussion phase, the adult guides the debate toward critical reflection on the results and the performance of the activity. This can be done through the formulation of questions, with the adult maintaining an objective stance and without issuing judgments or opinions. The program uses different techniques of group dynamics to enhance the development of the activity and the debate. Role-playing, brainstorming, and guided discussion through the formulation of questions are examples of these techniques.

### Data Analysis

To assess the effect of the program, firstly, descriptive analysis (means and SD) and univariate and multivariate analyses of variance (MANOVA, ANOVA) were conducted with the pretest scores of experimental and control adolescents. Secondly, descriptive and covariance analyses of the pretest–posttest differences were performed (MANCOVA, ANCOVA), with the pretest differences as covariates. Lastly, to assess the stability of the results, descriptive and covariance analyses of the pretest-follow-up differences were conducted (MANCOVA, ANCOVA), with the pretest differences as covariates. The effect size was calculated in each variable (Cohen’s *d*). In addition, the same analyses were performed comparing the pretest–posttest scores of the experimental boys and girls in the variables under study, in order to assess whether the change was similar in both genders.

## Results

### Effects of the Program on EI, Social-Conflict Resolution Strategies, Feelings of Anger, and the Ability to Analyze Negative Feelings

To assess the baseline in both conditions before the intervention, multivariate analysis on the pretest scores obtained on the instruments (TMMS-24, EIS, STAXI-NA, CECAS) was performed in the experimental and control groups. The results of the pretest MANOVA for the set of variables showed no differences between the experimental and control groups prior to the intervention, Wilks’ Lambda Λ = 0.913, *F*(15,132) = 0.84, *p* > 0.05. Descriptive analyses (mean and SD) of each variable and the results of the pretest ANOVAs (see **Tables 3** and **4**) indicate that, prior to implementing the program, there were no significant differences between the experimental and control groups in the assessed variables, showing the high level of homogeneity between the two conditions.

**Table 3:** Means and SD of the target variables [emotional intelligence (EI), social interaction strategies, anger, and cognitive ability to analyze negative feelings], in experimental and control groups, at pretest, in the pretest–posttest and pretest-follow-up differences.

	Pretest (*N* = 148)				Pretest–Posttest (*N* = 148)				Pretest-Follow-up (*N* = 130)			
	Experimental		Control		Experimental		Control		Experimental		Control	
	*M*	SD	*M*	SD	*M*	SD	*M*	SD	*M*	SD	*M*	SD
**Emotional intelligence**					
Attention	26.58	6.14	26.22	6.33	4.71	5.24	-1.05	7.19	2.43	6.83	-1.26	6.43
Clarity	26.16	6.04	26.18	6.39	4.96	5.69	-0.06	7.08	2.86	7.55	0.02	8.59
Repair	27.76	5.03	26.03	6.02	4.57	5.12	-0.03	5.66	3.24	5.96	0.05	6.30
**Social interaction strategies**					
Passive	3.55	2.58	3.29	2.47	-1.05	2.33	-0.57	2.55	-0.93	2.76	-0.21	2.83
Assertive	4.17	1.63	4.43	1.77	1.51	2.14	-1.29	1.42	1.57	2.20	-1.05	2.02
Aggressive	1.86	1.33	1.88	1.66	-0.37	1.65	-0.02	1.75	-0.46	1.77	0.29	1.56
**Feelings of anger**					
State-Anger	9.48	2.67	9.49	2.74	0.67	4.16	0.77	3.85	-0.42	3.18	0.40	3.50
Trait-anger	13.53	3.10	13.49	3.13	0.05	2.91	0.08	3.14	0.19	2.83	0.48	3.04
External expression	7.17	1.93	7.66	2.10	0.12	1.83	-0.46	2.27	0.17	2.16	-0.53	1.86
Internal expression	6.89	1.83	6.68	1.76	-0.67	1.68	-0.06	2.07	-0.65	2.00	-0.05	2.53
External control	8.22	2.33	8.05	2.05	0.51	2.12	-0.03	1.94	0.28	2.39	-0.05	2.18
Internal control	8.42	2.22	8.37	2.45	0.80	2.01	-0.38	2.78	0.40	2.76	-0.34	2.82
**Analysis of emotions**					
Causes	4.47	2.08	4.31	1.66	2.36	3.05	-0.22	2.21	1.65	2.86	0.00	2.56
Consequences	3.93	2.10	3.83	2.21	1.83	3.18	-0.45	2.84	1.99	3.21	0.40	2.78
Resolution	3.51	1.61	3.85	1.33	2.84	2.79	-0.80	1.97	2.22	3.35	-0.26	1.69

**Table 4:** Results of the pretest ANOVA, pretest–posttest ANCOVA, pretest-follow-up ANCOVA and effect size (*d*) of the experimental and control groups in the target variables (EI, social interaction strategies, anger, and cognitive ability to analyze negative feelings).

	Pretest			Pretest–Posttest			Pretest-Follow-up		
	*F*(1,146)	*p*	*d*	*F*(1,146)	*p*	*d*	*F*(1,128)	*p*	*d*
**Emotional intelligence**					
Attention	0.12	0.725	0.05	44.47	0.000	0.91	14.45	0.000	0.55
Clarity	0.00	0.978	-0.00	27.87	0.000	0.78	5.88	0.017	0.35
Repair	3.61	0.059	0.31	48.93	0.000	0.85	24.37	0.000	0.52
**Social interaction strategies**					
Passive	0.39	0.534	0.10	0.82	0.366	-0.19	1.76	0.187	-0.25
Assertive	0.87	0.352	-0.15	98.98	0.000	1.54	70.35	0.000	1.24
Aggressive	0.01	0.931	-0.01	3.08	0.081	-0.20	12.65	0.001	-0.45
**Feelings of anger**					
State-anger	0.00	0.982	-0.00	0.01	0.905	-0.02	2.57	0.111	-0.24
Trait-anger	0.00	0.942	0.01	0.02	0.867	-0.00	0.40	0.526	-0.09
External expression	2.19	0.141	-0.24	1.06	0.305	0.28	2.30	0.131	0.34
Internal expression	0.51	0.474	0.11	3.51	0.063	-0.32	1.24	0.266	-0.26
External control	0.21	0.643	0.07	3.37	0.068	0.26	1.27	0.262	0.14
Internal control	0.02	0.892	0.02	14.69	0.000	0.48	2.93	0.089	0.26
**Analysis of emotions**					
Causes	0.26	0.610	0.08	39.72	0.000	0.96	17.11	0.000	0.60
Consequences	0.07	0.786	0.04	26.33	0.000	0.75	12.73	0.001	0.53
Resolution	1.87	0.174	-0.23	84.97	0.000	1.50	26.93	0.000	0.93

Subsequently, to assess the effectiveness of the program on the variables under study, we analyzed the pretest–posttest change. The results of the MANCOVA carried out with the pretest–posttest differences in all the variables, with the pretest scores as covariates, revealed statistically significant differences between the experimental and control groups, Wilks’ Lambda Λ = 0.345, *F*(15,117) = 14.81, *p* < 0.001, with a large effect size (η^2^ = 0.655, *r* = 0.81). Regarding each variable, descriptive analyses (means, SD) and analysis of covariance of the pretest–posttest differences between the two conditions were conducted, with the pretest scores as covariates (see **Tables 3** and **4**). The results of the pretest–posttest ANCOVAs (see **Table 4**) confirmed statistically significant differences in the pretest–posttest change between the experimental and control groups.

In particular, we noted a significant increase in the mean scores of the experimental group (*Me*) compared with the control group (*Mc*) in EI, especially due to the significant improvement in its three factors, emotional attention (*Me* = 4.71, *Mc* = -1.05), emotional clarity (*Me* = 4.96, *Mc* = -0.06), and emotional repair (*Me* = 4.56, *Mc* = -0.03). Significant increases were also observed in assertive social-conflict resolution strategies (*Me* = 1.51, *Mc* = -1.29), internal control of anger (*Me* = 0.80, *Mc* = -0.38), and the ability to analyze the causes (*Me* = 2.36, *Mc* = -0.22) and consequences of negative feelings (*Me* = 1.83, *Mc* = -0.45), as well as in ways to positively cope with them (*Me* = 2.84, *Mc* = -0.80). Whereas the experimental group increased their scores in all the variables, the control group decreased theirs. The effect size was medium for internal control of anger and large for the other variables.

Subsequently, in order to assess the maintenance of the effects of the program 1 year after completing the intervention, the pretest-follow-up change was analyzed. The results of the MANCOVA on the pretest-follow-up differences in the set of assessed variables, with the pretest scores as covariates, showed statistically significant differences between the experimental and control groups, Wilks’ Lambda Λ = 0.453, *F*(15,99) = 7.97, *p* < 0.001, with a medium-high effect size (η^2^ = 0.547, *r* = 0.74). The results of the descriptive analysis (mean and SD) and pretest-follow-up ANCOVAs (with the pretest scores as covariates, see **Table 4**) confirmed statistically significant differences in the pretest-follow-up change between the experimental and control groups.

In particular, we noted a significant increase in the mean scores of the experimental group (*Me*) compared with the control group (*Mc*) in EI due to the significant improvement in all three factors, emotional attention (*Me* = 2.43, *Mc* = -1.26), emotional clarity (*Me* = 2.86, *Mc* = 0.02), and emotional repair (*Me* = 3.24, *Mc* = 0.05). Significant increases were also observed in assertive social-conflict resolution strategies (*Me* = 1.57, *Mc* = -1.05), the ability to analyze the causes (*Me* = 1.65, *M*c = 0.00), and consequences of negative feelings (*Me* = 1.99, *M*c = 0.40), as well as in constructive coping with these feelings (*Me* = 2.22, *Mc* = -0.26). In addition, we found a significant decrease in the use of aggressive social-conflict resolution strategies (*Me* = -0.46, *Mc* = 0.29). Although in the variable internal anger control, the experimental group increased its scores whereas the control group decreased theirs, the differences were not statistically significant. The effect size was large for assertive social interaction strategies and resolution of negative feelings, and medium in the other variables.

### Effects of the Program: Differences between Genders

The MANOVA on the pretest scores obtained in all instruments by the experimental boys and girls showed gender differences prior to the intervention, Wilks’ Lambda Λ = 0.612, *F*(15,67) = 2.83, *p* = 0.002, with a medium effect size (η^2^ = 0.388, *r* = 0.62). As shown in **Table 5**, the descriptive analyses (means and SD) and pretest ANOVAs show that, before the intervention, there were statistically significant gender differences in emotional attention, assertive social interaction strategies, state-anger, external control of anger, and identification of causes and consequences of negative feelings, with the girls obtaining higher scores in all variables except for external control of anger.

**Table 5:** Means, SDs, results of the pretest ANOVA, pretest–posttest ANCOVA, and effect size (*d*) in the studied variables (EI, social interaction strategies, anger, and cognitive ability to analyze negative feelings), in boys and girls.

	Pretest				Pretest–Posttest				Pretest ANOVA			Pretest–Posttest ANCOVA		
	Boys		Girls		Boys		Girls							
	*M*	SD	*M*	SD	*M*	SD	*M*	SD	*F*(1,81)	*p*	*d*	*F*(1,81)	*p*	*d*
**Emotional intelligence**					
Attention	24.32	5.47	28.39	6.09	6.92	5.34	2.93	4.48	9.98	0.002	-0.70	4.03	0.048	0.80
Clarity	25.78	6.14	26.46	6.01	6.03	5.62	4.11	5.66	0.25	0.617	-0.11	4.33	0.041	0.34
Repair	26.62	5.67	28.67	4.30	5.46	5.64	3.85	4.60	3.51	0.065	-0.40	0.94	0.335	0.31
**Social interaction strategies**					
Passive	3.51	2.58	3.59	2.61	-1.05	2.08	-1.04	2.54	0.01	0.898	-0.03	0.12	0.724	-0.00
Assertive	3.57	1.88	4.65	1.21	1.57	2.31	1.46	2.02	10.09	0.002	-0.68	4.37	0.040	0.05
Aggressive	1.92	0.95	1.80	1.58	-0.27	1.57	-0.46	1.73	0.15	0.700	0.09	1.25	0.266	0.11
**Feelings of anger**					
State-anger	10.19	3.42	8.91	1.69	-0.30	4.00	1.46	4.16	4.90	0.030	0.47	0.64	0.425	-0.43
Trait-anger	13.51	3.11	13.54	3.12	0.08	3.17	0.02	2.71	0.00	0.965	-0.01	0.67	0.414	0.02
External expression	6.76	1.84	7.50	1.95	0.00	1.81	0.22	1.87	3.11	0.081	-0.39	4.64	0.034	-0.12
Internal expression	6.76	1.92	7.00	1.77	-0.97	1.44	-0.43	1.83	0.35	0.552	-0.13	4.47	0.038	-0.32
External control	8.95	2.05	7.63	2.39	-0.05	1.91	0.96	2.19	7.00	0.010	0.59	0.78	0.379	-0.49
Internal control	8.11	2.14	8.67	2.27	0.78	1.88	0.80	2.12	1.33	0.251	-0.25	0.61	0.435	-0.01
**Analysis of emotions**					
Causes	3.84	1.66	4.98	2.26	2.05	2.65	2.61	3.34	6.54	0.012	-0.57	3.80	0.055	-0.18
Consequences	3.38	2.06	4.37	2.04	1.78	3.21	1.87	3.18	4.78	0.032	-0.48	2.30	0.133	-0.03
Resolution	3.16	1.42	3.78	1.72	2.97	3.07	2.74	2.57	3.09	0.082	-0.39	0.22	0.641	0.08

Subsequently, to assess whether the program differentially affected boys and girls, we conducted a MANCOVA of the pretest–posttest differences in all the assessed variables (with the pretest scores as covariates), the results of which revealed statistically significant differences in the change produced in both genders due to the effect of the intervention, Wilks’ Lambda Λ = 0.527, *F*(15,52) = 3.10, *p* = 0.010. The pretest–posttest ANCOVAs (see **Table 5**) confirm that—taking into account the variables in which the program was effective—the change due to the intervention in both genders was similar in most of the variables, except for emotional attention and clarity and assertive social-conflict resolution strategies. In these instances, boys improved significantly more than girls, although the effect size was small for all the variables except emotional attention.

## Discussion

The goals of this study were to assess the effects of an EI program on variables related to the prevention of violence, as well as to assess its differential effect in both genders. Firstly, the results have shown that the program significantly promoted an increase in EI (emotional attention, clarity, and repair), which was maintained at follow-up. Therefore, Hypothesis 1 is confirmed in its entirety. The findings confirm the results of other studies, indicating that these interventions increase EI (Di Fabio and Kenny, 2011) and emotional skills (Brackett et al., 2010).

An explanation of the positive effect of the program on EI may be related to the characteristics of the activities employed, as several of them promote intrapersonal intelligence (e.g., in “modifying beliefs” the students write out phrases about their own negative beliefs and transform them into positive phrases), interpersonal intelligence (e.g., in “emotions trivial pursuit,” the adolescents express emotions through gestures, drawings, and words so that their classmates can identify and understand them), or positive mood (e.g., in “feeling better,” the students dance around the classroom, listening to different styles of music that guide them to different positive moods).

Secondly, the results have shown that the program increased assertive social-conflict resolution strategies. These results were maintained at follow-up, and furthermore, the use of aggressive strategies as an interpersonal conflict-resolution technique was significantly reduced at this stage, confirming Hypothesis 2 almost in its entirety. These results point in the same direction as other studies that have shown the positive effect of socio-emotional intervention programs to increase assertive cognitive social interaction strategies (Garaigordobil, 2008), personal and social problem solving (Byrne et al., 2004), and to reduce anger and aggression (Castillo et al., 2013).

The fact that aggressive social interaction strategies decreased significantly only in the pretest-follow-up comparison may be due to the fact that it is easier to promote positive strategies than to reduce negative ones, the latter requiring more intervention time. In addition, the results show that the program not only promotes improvements between pretest and posttest, but continues to have an effect after completing the intervention. In part, this positive change can be explained because many activities of the program deal with conflict resolution and promote assertiveness. Some activities use role-playing of conflictive situations, encouraging students to seek assertive and non-violent solutions, whereas others present videos with problematic situations, and the group members should identify constructive ways to solve these conflicts.

Thirdly, the results obtained have confirmed that, between pretest and posttest, the intervention program showed a positive effect on internal anger control. Although anger control also increased between pretest and follow-up, the difference between the experimental and control groups was not statistically significant. Therefore, Hypothesis 3 is not confirmed. Although some studies show that socio-emotional interventions reduce anger, aggressiveness (Castillo et al., 2013) and disruptive behaviors (Cook et al., 2000; Flay et al., 2001), research assessing the effects of a program in which feelings of anger are scarce still confirm our results, which show that socio-emotional intervention programs improve self-control of behavior (Maurer et al., 2004).

These results may be partly explained because the activities of the program place considerable emphasis on assertiveness, peaceful conflict-resolution strategies, and empathy. In this way, the importance of avoiding violence is transmitted to adolescents, and they are taught to channel their feelings of anger, improve their anger control, and express emotions appropriately. However, the program places more emphasis on enhancing skills and positive traits than on reducing the negative ones. In fact, there is no activity that is aimed (directly) at reducing feelings of anger, but there are many activities that teach how to resolve disputes non-aggressively, avoiding anger expression in anger situations, though one activity does deal with self-control by means of a letter that the adolescents write to someone who has hurt them, talking about their feelings and recounting their version assertively.

Fourthly, the results confirm that the program had a positive effect by improving the ability to analyze the causes and consequences of negative emotions, as well as the ability to identify constructive ways of coping with these emotions. This effect was maintained at the follow-up phase, which confirms Hypothesis 4. Investigations of this variable confirm the results obtained in this study, as they support the ability of socio-emotional intervention programs to improve the capacity to analyze feelings (Garaigordobil, 2008).

The program improves the ability to analyze emotions and to cope with them positively, because it contains many activities that encourage the adolescents to analyze the causes, factors or stimuli that generate negative emotions, the negative consequences of such emotions (for the person who feels those emotions and for others), and ways to cope positively with negative emotions (e.g., through positive dialog, symbolization, etc.). For example, in the activity called “emotional perception,” participants watch videos and answer questions about the causes and consequences of the emotions of the video characters. In another activity, “collage and dramatization,” the students make a collage telling a story, which expresses an emotion, with a beginning, a problem, and an ending.

Fifthly, Hypothesis 5 is confirmed almost in its entirety, because the change produced by the intervention in both genders was similar, except for the variables emotional attention/clarity and assertive social interaction strategies, in which boys improved more than girls. These results are consistent with those obtained in studies that have generally shown a similar change due to the effect of the intervention on boys and girls (Eisen et al., 2003; Kimber et al., 2008), but also with studies that have shown that boys improve EI (Melero and Palomera, 2011), and empathy (Castillo et al., 2013) the most. The fact that the boys have increased their scores significantly more due to the program may be because at pretest, they had a lower level in these variables and, therefore, they had more room for improvement.

Finally, the data have confirmed that the positive effects of the program were maintained 1 year after completing the intervention in all variables except for internal anger control. In addition, the use of aggressive interpersonal conflict-resolution strategies decreased significantly more in the experimental group at follow-up. As already noted in previous studies (Garaigordobil, 2008; Garaigordobil and Martínez-Valderrey, 2014), this might be due to the fact that during adolescence, it is easier to promote positive behaviors and strategies than to decrease the negatives ones, which certainly requires more time. Therefore, Hypothesis 6 is verified almost in its entirety, adding support to findings of other studies that conducted a follow-up and found some maintenance of the effects of the intervention (Byrne et al., 2004; Ruiz-Aranda et al., 2012).

The quantitative results point in the same direction as the qualitative information provided by the adolescents at the end of the intervention. In the debate of the last session, the question, “what have you learned?” produced answers such as: I have learned: “to relax and now I sleep better,” “now I know what empathy is” “to be aware that other people feel differently and to better understand other people,” “to control my feelings, for example, the other day I got very angry at a classmate and when I was going to hit her, I remembered what I had learned in the program and I didn’t hit her,” “to respect people more,” “different ways to express emotions,” “to know emotions better,” “to relate better to classmates; before, I had no friends and now, I’m in a group.” This qualitative information is consistent with the quantitative results obtained in the present study.

This study has practical implications for the psycho-educational field, providing an evidence-based tool to develop EI and reduce variables related to violence. The positive change produced by the program can be explained by the characteristics of the activities it contains and also by the relevant meta-cognitive role of the discussion phase. The results of the assessment of the program allow us to suggest the importance of implementing programs that promote social-emotional development during childhood and adolescence, as a prevention and intervention tool (Garaigordobil and Fagoaga, 2006). In addition, the scientific evidence of the efficacy of emotional education programs is quite limited (Pérez-González, 2012), so that this study makes a significant contribution to psycho-educational research. Moreover, it should be noted that it would be beneficial to integrate intervention programs as a part of school education.

There are multiple arguments to justify emotional education, because currently, a large percentage of adolescents and young people are involved in violent behavior (bullying, cyberbullying, sexism, self-inflicted violence, etc.) and in risk behavior (drug consumption, etc.) (World Health Organization [WHO], 2014).

These problems are emotion-based, and as such, the need to implement prevention programs that affect multiple behaviors (violence, drug use, stress, depression, etc.) is obvious. But in addition to prevention, it is important to build well-being proactively. Therefore, a solution to this problem may be the implementation of programs that develop social skills and emotional competence. In fact, the effectiveness of such programs has been demonstrated in several studies. For example, a meta-analysis by Durlak et al. (2011) assessed 213 prevention programs used in schools (from kindergarten to secondary school) and showed the positive effects of these programs in increasing personal and social skills, mental health, and positive development, at the same time they decreased antisocial behavior, substance abuse, aggressiveness, and disciplinary problems.

A limitation of the study is the use of self-reports with the social desirability biases involved, so we recommend future studies to replicate the investigation using assessment instruments completed by parents/teachers and/or observational methodology. As future lines of research, we suggest the assessment of the effects of the program on other variables (self-esteem, empathy, psychopathological symptoms, etc.), as well as increasing the involvement of the schools (for example, through the training of teachers) and parents (for example, through the parents’ schools) in the process of developing EI in order to improve the results of the intervention.

## Conflict of Interest Statement

The authors declare that the research was conducted in the absence of any commercial or financial relationships that could be construed as a potential conflict of interest.
